# Ovarian Torsion Secondary to a Mature Cystic Teratoma in a Four-Year-Old Girl: A Case Report

**DOI:** 10.7759/cureus.99561

**Published:** 2025-12-18

**Authors:** Fatima Smahi, Hind Cherrabi, Zineb Benmassaoud, Mohammed Eljadid, Mohamed Amine Oukhouya

**Affiliations:** 1 Pediatric Surgery, Souss Massa University Hospital, Agadir, MAR; 2 Pediatric Surgery, Hospital Hassan II, Agadir, MAR

**Keywords:** child, mature teratoma, ovarian mass, surgery, torsion

## Abstract

Teratomas are the most common histological subtype of germ cell tumors in pediatric patients. They are classified into two types: mature(benign) and immature(malignant). Early diagnosis is essential for optimal management. Adnexal torsion is the most frequent complication of mature ovarian teratoma, with a higher incidence in young girls. We report a case of a four-year-old girl who arrived at the emergency department with acute left flank abdominal pain and non-bloody vomiting, 24 hours prior to admission. Abdominal ultrasound revealed a large cystic abdominal mass with a Doppler-detected vascularized spiral configuration. Surgical management was performed via a Pfannenstiel laparotomy, which revealed a mature ovarian teratoma.

## Introduction

Germ cell tumors are the most common ovarian neoplasms in children and adolescents, and teratomas are the most frequent among them [1]. Teratomas arise from pluripotent germ cells and contain elements from all three germ layers; they are classified as mature or immature [2]. A mature cystic teratoma (dermoid cyst), composed of well-differentiated tissues derived from the ectoderm, mesoderm, and endoderm, is one of the most common benign ovarian tumors [3,4].

We report the case of a four-year-old girl who presented with acute abdominal pain secondary to ovarian torsion on a mature ovarian teratoma. This case is noteworthy because it involves ovarian torsion secondary to a giant mature cystic teratoma in a very young, prepubertal girl, with a prolonged duration of symptoms before diagnosis. These features make the case clinically challenging and highlight the risk of delayed recognition and loss of ovarian function.

## Case presentation

A four-year-old girl, with no notable pathological history, presented to the emergency department with acute abdominal pain localized to the left flank, with non-bloody vomiting 24 hours prior to admission. Clinical examination revealed an apyretic patient in good general condition with a distended abdomen. Palpation revealed a left flank mass with pelvic tenderness.

**Figure 1:**
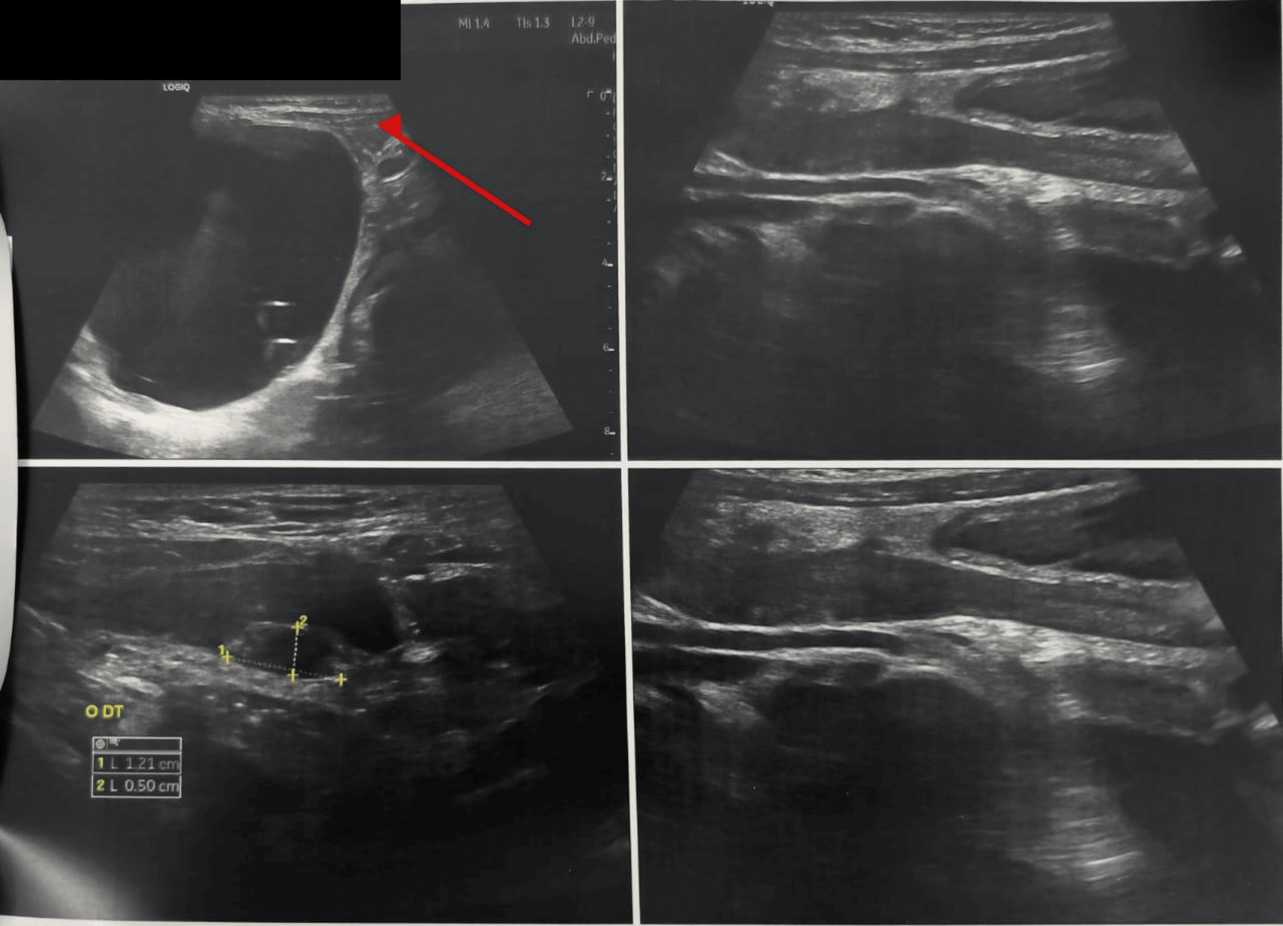
Abdominal ultrasound showing a large cystic abdominal mass occupying most of the abdominal cavity, suggestive of an adnexal origin (red arrow) Red arrow: cystic mass on the left side

Abdominal ultrasound revealed a large abdominal cystic mass with heterogeneous internal echoes (Figure 1). Color Doppler interrogation demonstrated a twisted, vascularized pedicle with the characteristic ‘whirlpool sign’, highly suggestive of left adnexal torsion. The left ovary was difficult to visualize, whereas the right ovary appeared normal.

**Figure 2:**
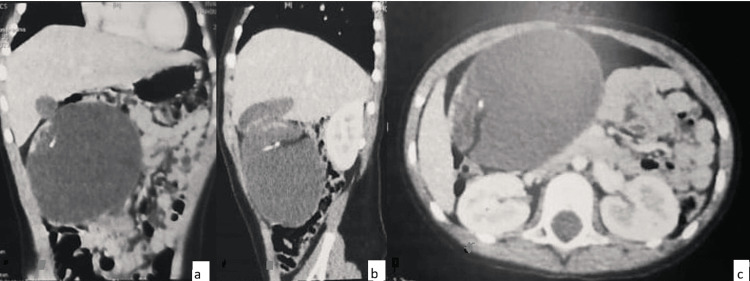
CT scan showing coronal (a), sagittal (b), and axial (c) sections revealing a cystic mass on the left side measuring 90x76x98 cm, the presence of a fatty component, calcium, and a few thin superolateral right septa

A CT scan of the abdomen and pelvis revealed a cystic mass on the left flank measuring 90 × 76 × 98 mm. The lesion had a thin wall and heterogeneous contents, with fatty and calcified components. A few fine superolateral right septations were observed, probably corresponding to hemorrhagic recurrences (63 HU density) without contrast enhancement. A dense band containing vascular structures extended from the inferomedial surface of the mass to the uterine fundus, consistent with a twisted pedicle (Figure 2).

**Figure 3:**
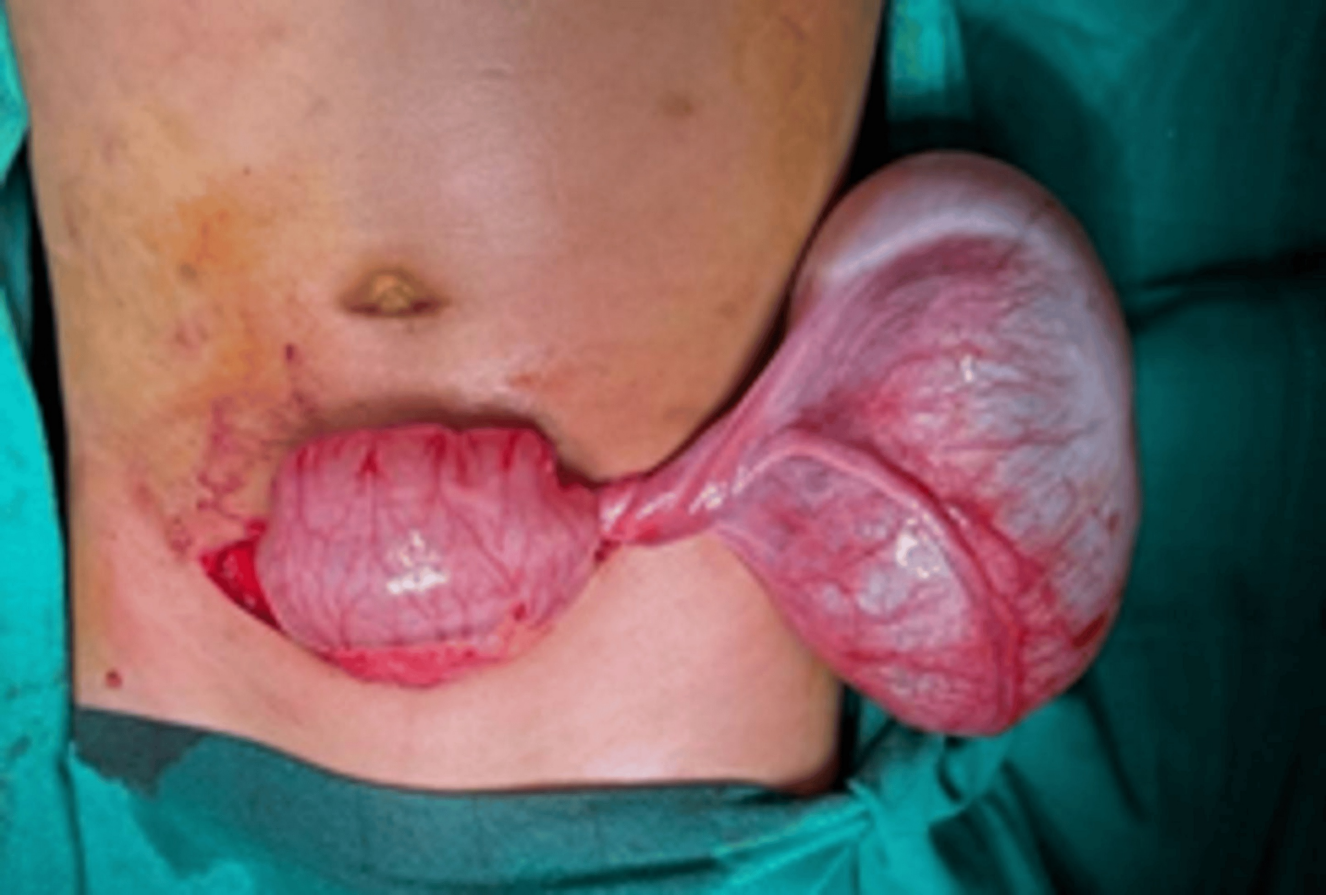
Preoperative image showing the twisted appearance of the left ovarian mass

Biological tests revealed that serum α-fetoprotein (AFP) and β-hCG levels were negative. The patient underwent a Pfannenstiel laparotomy. Intraoperatively, a large solid mass arising from the left ovary was identified, associated with a markedly stretched and twisted fallopian tube forming a spiral configuration (Figure 3).

**Figure 4:**
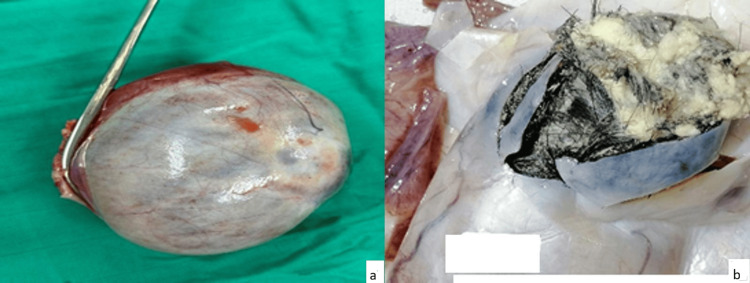
Macroscopic appearance of the ovarian mass showing a smooth external surface (a) and the presence of hair and sebaceous material within the mass (b)

**Figure 5:**
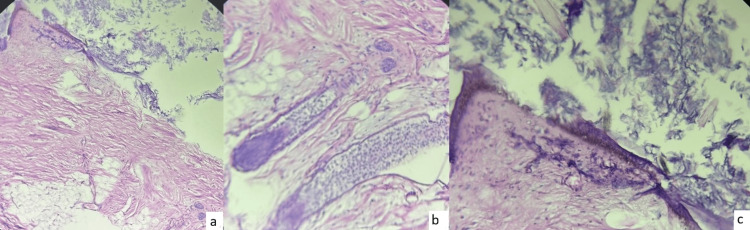
Histological sections showing (a) an epidermal lining with keratin on the surface, (b) the presence of a few hair follicles, and (c) keratin associated with hair shafts

There were no signs of perforation of the lesion and no adhesions with adjacent organs. Exploration of the remainder of the abdominal and pelvic cavity was unremarkable. Given the torsion and non-viability of the adnexa, a left adnexectomy was performed. Histological analysis of the mass showed an epidermal lining with surface keratin; the lumen was filled with hair shafts, and a few hair follicles were also identified (Figures 4, 5). These findings were consistent with a mature teratoma of the left ovary.

The postoperative course was uneventful. The patient was discharged on postoperative day 2, and a follow-up ultrasound performed at one month was normal, showing a right ovary of normal size and no peritoneal effusion.

## Discussion

Teratomas are classified as either mature or immature [5]. In children, mature cystic teratomas are the most frequent ovarian tumors, accounting for up to 20% of ovarian neoplasms [6]. Bilaterality is observed in approximately 12% of cases, and the risk of malignancy is estimated at about 2% [7]. Adnexal torsion, although uncommon, is the main complication of mature ovarian teratomas in the pediatric population [8]. Clinical presentation is highly variable and nonspecific, ranging from abdominal pain to digestive or pelvic symptoms [9].

Ultrasound with a full bladder is the first-line imaging modality in children, allowing assessment of the lesion, its location, and associated abnormalities. Plain abdominal radiography is not routinely recommended but may occasionally reveal calcifications or ossifications [10]. When malignancy is suspected or the diagnosis remains uncertain, additional imaging is required. On ultrasound, mature teratomas typically appear as unilateral, unilocular cystic masses containing echogenic components corresponding to fat, hair, calcifications, or ossified structures [11,12]. These features are consistent with the classic radiological description of mature cystic teratomas, although atypical presentations, particularly in prepubertal girls or in large lesions, can complicate diagnosis.

Histological analysis remains essential to confirm the diagnosis and guide management. Sonographic features suggesting malignancy are variable and nonspecific [13-15]. In such cases, CT and MRI are invaluable, as they are more sensitive in detecting intralesional fat and assessing associated complications [16,17]. Although ovarian teratomas are usually non-secretory [18], the measurement of tumor markers is systematic to exclude malignant germ cell tumors. Elevated α-fetoprotein suggests a yolk sac tumor, whereas elevated β-hCG indicates a choriocarcinoma.

Macroscopically, mature teratomas usually appear as unilocular cysts filled with sebaceous material, keratinous debris, and hair [11]. In contrast, immature teratomas are typically large, encapsulated, and predominantly solid, often with irregular calcifications and multiple fat foci [12,17]. Histologically, they are distinguished from mature teratomas by the presence of immature tissue, and the proportion of immature elements determines the tumor grade [11,12]. Immature teratomas may coexist with a homolateral mature teratoma in 26% of cases and with a contralateral one in 10% [12].

Recent pediatric work has emphasized risk-stratified management of benign ovarian neoplasms, using consensus-based preoperative algorithms to limit unnecessary oophorectomies while reserving surgery for masses with symptoms or higher-risk features [13]. Contemporary pediatric studies and a recent meta-analysis support ovarian-sparing surgery as the preferred approach for benign ovarian tumors whenever viable tissue is present, with high ovarian preservation rates and low failure of therapy after cystectomy or tumorectomy [16]. Oophorectomy or adnexectomy is required when ovarian tissue cannot be preserved, in cases of torsion, or when there is a risk of malignancy, including positive tumor markers [19]. Immature teratomas behave more aggressively, with potential locoregional spread, and treatment consists of unilateral adnexectomy, often combined with chemotherapy depending on the stage [17].

The prognosis of mature ovarian teratomas is excellent. However, there is a 3-13% risk of metachronous lesions in the contralateral ovary, which does not always require surgical intervention [9,12,14-16]. Recent large pediatric cohort data report high ovarian preservation rates after ovary-sparing surgery and low recurrence rates of benign ovarian tumors, and support structured postoperative ultrasound follow-up for several years to detect ipsilateral recurrence or contralateral metachronous disease [20]. Most authors recommend ultrasound surveillance rather than routine tumor marker monitoring after surgery when preoperative markers are normal and the lesion has been completely excised [20].

## Conclusions

Ovarian torsion is a rare but serious condition in children that can progress from ovarian edema to hemorrhagic infarction, culminating in total necrosis and compromised fertility if not treated promptly. It should always be considered in the differential diagnosis of acute abdominal pain in young girls, as early surgical intervention is critical for preserving ovarian health and future reproductive function.

This case highlights that ovarian torsion secondary to a mature cystic teratoma can occur even in very young prepubertal girls and may present as a giant left ovarian mass with characteristic imaging findings, including the whirlpool sign and a twisted adnexal pedicle. Recognizing these specific features can facilitate timely diagnosis and appropriate surgical decision-making, balancing ovarian preservation with oncological safety.
